# Editorial: Novel Technological and Methodological Tools for the Understanding of Collective Behaviors

**DOI:** 10.3389/frobt.2019.00139

**Published:** 2019-12-10

**Authors:** Elio Tuci, Vito Trianni, Andrew King, Simon Garnier

**Affiliations:** ^1^Faculty of Informatics, University of Namur, Namur, Belgium; ^2^Institute of Cognitive Sciences and Technologies, Italian National Research Council (CNR), Rome, Italy; ^3^Department of Biosciences, Swansea University, Swansea, United Kingdom; ^4^Department of Biological Sciences, Institute for Communities and Wildlife in Africa, Cape Town, South Africa; ^5^Department of Biological Sciences, New Jersey Institute of Technology, Newark, NJ, United States; ^6^Department of Biological Sciences, Rutgers University, Newark, NJ, United States

**Keywords:** collective behavior, self-organization, emergence, collective dynamics, tools and methods

## 1. Introduction

The social processes that give rise to coordinated actions of a group of agents and the emergence of global structures—referred to as collective behaviors—are observed in a range of biological and artificial systems. Collective behavior research, therefore, focuses upon a range of different phenomena with the common goal of understanding the dynamics of emergent group level responses, and has resulted in a burgeoning, diverse, and interdisciplinary research community.

Studying collective behaviors in biological and artificial systems is particularly challenging because of their intrinsic complexity, requiring novel approaches that can help unraveling these systems in order to explain how and why certain patterns are produced and maintained. This Research Topic brings together a collection of studies that focus on technological and methodological tools that can support the understanding of collective behaviors. The contributions included within the Research Topic can be broadly categorized as: (i) Review Articles, (ii) Tools and Technologies, and (iii) Empirical Studies.

Our goal is to facilitate the dissemination of ideas, theories, and methods among scientists that share an interest on the study of collective behavior in all its diverse manifestations. It is our hope that, together, this Research Topic and contributions may afford a more complete understanding of the nature of proximate and ultimate causes of collective behaviors in biological systems, and provide opportunity to generate a theoretical framework to engineer robust, resilient, and effective technologies, such as multi-robot systems, smart grids, and sensor networks.

## 2. Review Articles

Four review articles illustrate the state-of-the-art in the analysis of social dynamics in different research domains.

In Laan et al., the authors review different methodologies to aggregate individual information and restore the collective wisdom when simple averages are not sufficient, explaining when each methodology is applicable to real-world situations. The authors shows that advanced averaging procedures of the opinions of the members of a large crowd can lead to incredibly accurate collective decisions. However, this accuracy is highly context-dependent and relies on conditions that are often not realistic in practice.

In Moussaïd et al., the authors provide a thorough review of the use and potential for virtual reality and multi-user platforms for new types of experiments in crowd behaviors and describe how these new technologies can transform the way crowd research is conducted. Understanding human crowd dynamics can help urban planners manage crowd safety, ultimately preventing crowd disasters and saving lives.

In Bredeche et al., the authors review research studies focused on a methodology, called embodied evolution, used to design controllers for a group of robots characterized by the use of evolutionary computation techniques in an online fashion. That is, the evaluation, selection, and reproduction cycle runs in a decentralized way on each robot of the group while they are carrying out their task. The authors review a large body of relevant literature and point to a number of open issues that, from their perspective, need to be addressed to further develop this research field into a mature design methodology.

In Tuci et al., the authors provide a comprehensive summary of goals and objectives of the literature on cooperative transport in multi-robot systems. In cooperative transport, a group of robots is required to cooperate in order to transport objects that, due to their mass, shape, or size cannot be transported by single robots. The authors provide an interesting framework to organize a relatively heterogeneous body of work by using the transport strategy as a criterium to classify and sort the research works.

## 3. Tools and Technologies

Five articles illustrate new methodological tools for the study of collective behaviors.

In Moore et al., the authors illustrate the characteristics of a new library for efficient information-theoretic analysis of collective behaviors. The library proves to be computationally very efficient, inclusive of a larger set of information theoretic measures, and equipped with a suite of wrappers for higher-level programming languages that aim to make it accessible to a wide user-base.

In Boenisch et al., the authors describe a tool to track the movements of bees called BeesBook. Understanding collective behavior of natural systems requires powerful tools to determine the way in which individuals in the collective move and interact with each other. While several tracking softwares are being developed that allow to follow movements and interactions among several animals in a group, few approaches exist for long-term identity-based tracking of individuals. The BeesBook system has been deployed to follow every bee in a colony along a period of several weeks, tracking the movement and interaction of individual insects throughout their whole lifetime.

In Jones et al., the authors illustrate the Xpuck platform to analyse the behavior of a swarm of robots. The ability to synthesize relevant collective behaviors in robot swarms is often bound by the limited computational abilities of robotic platforms, which do not allow complex information processing (e.g., image analysis for machine vision), or advanced reinforcement learning techniques. The Xpuck platform is therefore an interesting proposal for experimental studies requiring large computational power on the swarm robots, coupling the miniature size of the e-puck platform with the computational power of modern GPUs.

In Nemitz et al., the authors propose a new robotic platform called HoverBot that offers increased functionality while maintaining production costs low. Designed around an innovative locomotion mechanism combining air levitation and magnets, the HoverBot is composed of a single PCB with no mechanical parts making it easy to mass produce and extend with new sensors. This new platform opens up interesting opportunities to implement collective intelligence algorithms on large robotics swarms.

In Bottinelli and Silverberg, the authors describe how methods developed to study granular materials can be applied to difficult-to-analyse patterns of collective motion in biological systems in high density conditions. The authors provide a step-by-step protocol for researchers to create “eigenmodes.” These eigenmodes identify hidden long-range motions and localized rearrangements of particles (or any social unit) based solely on their trajectories. This novel approach appears to be a promising new tool for identifying different types of emergent collective motion in biological systems.

## 4. Empirical Studies

Six articles illustrate results of new experiments focused on collective behavior.

In Hamann, the author study opinion dynamics in a group of mobile robots. A common problem associated with opinion dynamics models when adapted to physical systems—be they natural or artificial—is that the spatial distribution of the agents and their mobility result in spatial correlations that contrast the well-mixed assumptions at the basis of many macroscopic models, making them inappropriate to describe the overall system dynamics. An interesting intuition to grasp the effects of spatial correlation is to include a number of “contrarians” in the population, that is, agents that prefer the minority opinion. With such expedient, the author shows that macroscopic models can be tuned to match the dynamics shown by systems affected by spatial correlations.

For collective behavior studies, a very important ability is the precise identification of leaders in animal groups, as well as the dynamical aspects related to how leaders influence the group movements and how leadership changes from time to time in response to external events. Multiple methods have been proposed in the past, each with its own advantages and drawbacks. In Mwaffo et al., the authors show that a model-free methodology to combine existing methods in a maximum likelihood sense provides an invaluable tool for collective behavior research, allowing to robustly identify leaders from raw positional data.

Object retrieval and gathering has been a hallmark of swarm robotics since its inception in the 1990's. In Strömbom and King, the authors revive this concept using an algorithm derived from the behavior of sheepdogs. This algorithm uses a feedback loop between a video tracking system and a robot to control the robot movements in relationship to the objects to gather. Results show that the robot can efficiently collect and transport an object to a target location and, more importantly, can adapt its behavior to changing conditions and is robust to noise. This approach to object gathering offers interesting new perspectives for automated swarms of robots.

The use of robots in collective behavior research is a burgeoning area of research. If robots are “accepted” as conspecifics, they allow for experimental manipulations of social interactions. In Bierbach et al., the authors use a bio-mimetic fish in behavioral experiments with surface- and cave-dwelling fish (Poecilia mexicana). They found that both cave- and surface-dwelling fish followed and interacted with the robot when tested in light. However, when tested in darkness, only surface-dwelling fish were attracted to the robot in darkness suggesting the robot fish-replica is providing mostly visual cues. Such work is important because it determines (for this fish system) the mode of feedback between fish and robot, so that the robot (and thus fish) can become controllable by the experimenter.

Nonapeptides (NP) are neurohormones that are known to affect the performance and maintenance of various behaviors in animals, including partner and group preferences. In Ondrasek et al., the authors hypothesized that NP systems may be important mediators to avian collective behaviors, and mapped the distribution of NP receptors in the brain tissue of three flocking bird species—house sparrows, European starlings, and rock doves. The lateral septum, a brain area known to regulate avian flocking was found to have lots of NP receptors in all three species, and in sparrows and starlings the dorsal arcopallium was important too; an area of the brain that we know little about with respect to social behaviors or flocking. Ondrasek et al.'s findings provide an important first step toward the undertaking of neuroecological studies of collective behaviors in birds.

Statistical methods mediated from collective behavior research can be applied also to criminology. The distribution of criminal activities is influenced by the characteristics of the urban environment, but also by indications that a location is (or not) associated with past crimes. In Garnier et al., the authors combine Risk Terrain Modeling, a statistical tool to estimate the relationship between features of the urban environment and crime occurrences, and a model of the spatio-temporal dependence between successive criminal events to predict robberies in a large urban centre. They demonstrate that this two-pronged approach significantly improves upon state-of-the-art methods for predictive policing.

## 5. Conclusions

As the reader can clearly notice, the wide range of topics covered in this collection highlights the variety of the research in collective behaviors. The field is largely multi-disciplinary and always crosses the borders of single research domains. As a consequence, it strongly needs new opportunities for gathering together the multiple advances that are constantly proposed—with studies departing from different disciplines—in order to foster cross-fertilization and progress toward a shared understanding of collective behaviors. This Research Topic represents an attempt to provide such a ground: it focused on tools and methods that sometimes have been intended for a specific case (e.g., in the study of animal behavior) but that can be easily generalized to others (e.g., for the design and analysis of robot swarms). This is a pattern that has been followed many times in the past, and we hope that the research work presented here can be of inspiration for further developments in the future.

## Author Contributions

All authors listed have made a substantial, direct and intellectual contribution to the work, and approved it for publication.

### Conflict of Interest

The authors declare that the research was conducted in the absence of any commercial or financial relationships that could be construed as a potential conflict of interest.

